# Quasi‐Paired Pt Atomic Sites on Mo_2_C Promoting Selective Four‐Electron Oxygen Reduction

**DOI:** 10.1002/advs.202101344

**Published:** 2021-07-14

**Authors:** Lei Zhang, Tong Yang, Wenjie Zang, Zongkui Kou, Yuanyuan Ma, Moaz Waqar, Ximeng Liu, Lirong Zheng, Stephen J. Pennycook, Zhaolin Liu, Xian Jun Loh, Lei Shen, John Wang

**Affiliations:** ^1^ Department of Materials Science and Engineering National University of Singapore Singapore 117574 Singapore; ^2^ Department of Physics National University of Singapore Singapore 117551 Singapore; ^3^ State Key Laboratory of Advanced Technology for Materials Synthesis and Processing Wuhan University of Technology Wuhan 430070 P. R. China; ^4^ Institute of Materials Research and Engineering Agency for Science, Technology and Research (A*STAR) 2 Fusionopolis Way Singapore 138634 Singapore; ^5^ Beijing Synchrotron Radiation Facility Institute of High Energy Physics Chinese Academy of Sciences Beijing 100049 China; ^6^ Department of Mechanical Engineering National University of Singapore Singapore 117575 Singapore

**Keywords:** cleavage of O─O bond, electrocatalysts, oxygen reduction reaction, quasi‐paired Pt atoms, single atom catalysts

## Abstract

Atomically dispersed Pt species are advocated as a promising electrocatalyst for the oxygen reduction reaction (ORR) to boost noble metal utilization efficiency. However, when assembled on various substrates, isolated Pt single atoms are often demonstrated to proceed through the two‐electron ORR pathway due to the unfavorable O─O bond cleavage thermodynamics in the absence of catalytic ensemble sites. In addition, although their distinct local coordination environments at the exact single active sites are intensively explored, the interactions and synergy between closely neighboring single atom sites remain elusive. Herein, atomically dispersed Pt monomers strongly interacting on a Mo_2_C support is demonstrated as a model catalyst in the four‐electron ORR, and the beneficial interactions between two closely neighboring and yet non‐contiguous Pt single atom sites (named as quasi‐paired Pt single atoms) are shown. Compared to isolated Pt single atom sites, the quasi‐paired Pt single atoms deliver a superior mass activity of 0.224 A mg^−1^_Pt_ and near‐100% selectivity toward four‐electron ORR due to the synergistic interaction from the two quasi‐paired Pt atom sites in modulating the binding mode of reaction intermediates. Our first‐principles calculations reveal a unique mechanism of such quasi‐paired configuration for promoting four‐electron ORR.

## Graphical abstract

Quasi‐paired Pt atoms, distinguished by closely‐neighboring and yet non‐contiguous Pt single atoms acting in synergy, are stabilized on a mesoporous Mo_2_C support. The Pt_quasi_/Mo_2_C exhibits much superior selectivity and mass activity toward four‐electron oxygen reduction pathway as compared to both isolated Pt single atoms and Pt nanoparticles, owing to the unique O─O activation mechanism enabled by the quasi‐paired Pt atoms.

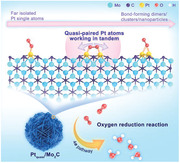

## 1 Introduction

Recently, in the extensive quest for higher precious metal utilization efficiency and boosting the catalytic mass activity and selectivity, single atom catalysts (SACs) have been explored vigorously. By downsizing the active materials into the atomic regime, SACs are expected to bring about unprecedented changes in catalytic properties by bridging the advantages of both homogeneous and heterogeneous catalysts.^[^ ^1^, ^2^, ^3^, ^4^ ^]^ As the atomically dispersed active metal centers are uniformly coordinated by atoms from the support material, one prominent feature in SACs is their well‐defined structural homogeneity and precisely‐tunable local coordination environment, which can hardly be achieved in nanoparticle/nanocluster‐based catalysts.^[^ ^5^ ^]^ Therefore, SACs have been widely advocated as ideal candidates for mechanistic studies and to judiciously tune the activity and selectivity toward certain catalytic reactions. Nevertheless, SACs may not be a panacea for all catalytic reactions, especially not for those that require multiple neighboring active atoms to work in tandem.^[^ ^6^, ^7^ ^]^ The absence of such ensemble sites in SACs often compromises the activity and/or selectivity toward certain reactions, such as the low‐temperature oxidation of CO, C_3_H_6_, and C_3_H_8_,^[^ ^8^, ^9^ ^]^ vinyl acetate synthesis,^[^ ^10^ ^]^ selective conversion of acetylene to ethylene,^[^ ^11^ ^]^ and ethylene hydrogenation.^[^ ^12^ ^]^

In particular, the oxygen reduction reaction (ORR) via the four‐electron (4*e*) pathway to form water requires cleaving the O─O bond, which can be greatly facilitated in the presence of ensemble sites, typically those on nanoparticles.^[^ ^13^ ^]^ Being a key half‐reaction in several next‐generation energy storage systems such as metal–air batteries and polymer electrolyte membrane fuel cells, the efficient and selective execution of the 4*e* ORR is essential in promoting high energy density and preventing corrosion to the separator membranes (caused by the production of H_2_O_2_ from the two‐electron ORR pathway).^[^ ^14^, ^15^ ^]^ Platinum (Pt)‐based catalysts represent the most widely explored ORR catalyst and the state‐of‐the‐art performance benchmark.^[^ ^16^ ^]^ However, in recent endeavors to develop atomically dispersed Pt catalysts to maximize noble metal utilization, many have demonstrated a high selectivity toward the 2*e* ORR instead, and concluded that isolated Pt single atoms cannot effectively break the O─O bond and therefore exhibit poor selectivity toward the 4*e* ORR process due to the absence of Pt–Pt ensemble sites.^[^ ^7^, ^13^, ^17^ ^]^

Currently, most of the Pt SACs rely on the strategy of having large‐surface‐area support materials coupled with relatively minute loadings of Pt to realize a stable dispersion of the single atoms. Therefore, the active Pt atoms are far isolated from their neighboring sites and assumed to have negligible interaction. Various attempts to study and tune the energy barrier and reaction pathways so far have focused on examining and tailoring the local coordination environment at the exact sites of the active centers, but only very few studies have expanded the exploration to involve synergistic interactions among the neighboring active sites.^[^ ^10^, ^18^ ^]^ In view of the above‐mentioned challenges and the large unfilled gap, it would be worth examining the interactions between closely spaced and yet non‐contiguous single atoms, to exploit their synergy in enhancing the intrinsic activity and selectivity of heterogeneous electrocatalysts.

Molybdenum carbides are promising support materials for anchoring Pt single atoms, owing to the especially strong metal–support interaction, and importantly their ability to anchor Pt single atoms in a wide concentration range thus allowing the modeling of potential synergistic interactions among the densely populated Pt single atoms without compromising the dispersion and stability.^[^ ^19^, ^20^, ^21^ ^]^ Herein, by taking ORR as a model reaction, and atomically dispersed Pt supported on a mesoporous orthorhombic molybdenum carbide (*β*‐Mo_2_C) substrate as a model catalyst, we examine the evolution of the ORR activity and mechanism with reference to the degree of separation between the neighboring Pt atoms by both experimental and computational studies. Catalysts with various Pt loadings were synthesized and their application in ORR was investigated. We observed a distinct switch in mass activity and selectivity being when the Pt loading was increased while the single atom dispersion was still maintained; we term this case the “quasi‐paired single atoms” in that two closely neighboring and yet non‐contiguous Pt sites exhibit synergistic interactions while remaining “single” (not directly bonded). The catalyst with quasi‐paired Pt single atoms demonstrates superior 4*e* ORR activity and selectivity as compared to its far isolated single atomic counterpart, exemplifying an optimum balance between single atom dispersion and synergy. By density functional theory (DFT) calculations, we further investigated the effect of mean Pt–Pt distance on the binding modes of reaction intermediates, and the theoretical overpotentials toward the 2*e* and 4*e* ORR pathways, to unveil the fundamentally different ORR selectivity of isolated, quasi‐paired, and paired Pt sites, respectively.

## 2 Synthesis and Characterization of Catalyst Materials

**Figure 1:**
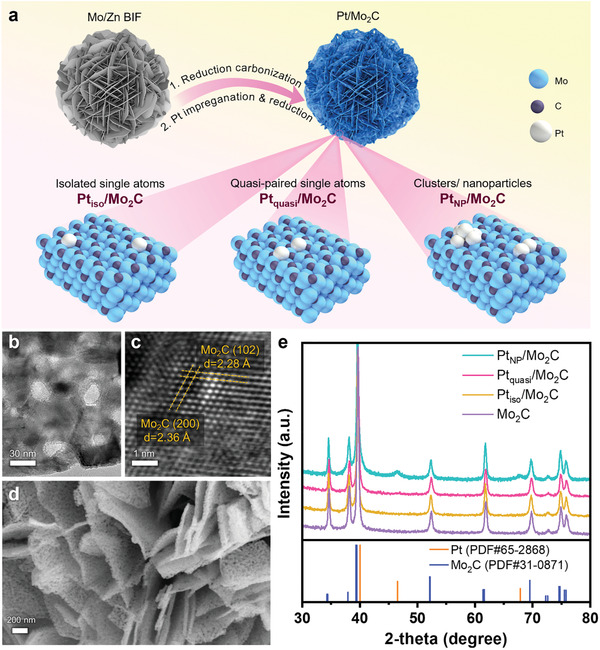
Schematic diagram and structural and phase characterization. a) Schematic diagram of the materials synthesis. b,c) TEM images of the *β*‐Mo_2_C support. d) SEM image of Pt_quasi_/Mo_2_C. e) XRD patterns of the *β*‐Mo_2_C support and various Pt/Mo_2_C samples.

As illustrated in **Figure** **1**a, during the synthesis, a Mo/Zn bimetallic imidazole framework (Mo/Zn BIF) is first formed as a precursor, and is then converted into Mo_2_C by heat treatment in a reducing atmosphere at 800 °C, during which Zn vaporizes, leaving behind a mesoporous structure as the catalyst support, which was reported in our previous work.^[^ ^22^ ^]^ With the incipient wetness impregnation (IWI) method, Pt/Mo_2_C samples with different mean Pt–Pt distances are synthesized by controlling the Pt mass loading, and the Pt contents are confirmed by inductively coupled plasma optical emission spectrometry (ICP‐OES). As a result, the samples with isolated Pt single atoms (Pt_iso_/Mo_2_C), quasi‐paired Pt single atoms (Pt_quasi_/Mo_2_C), and Pt nanoparticles with contiguous Pt sites (Pt_NP_/Mo_2_C) are obtained for comparison investigations, and their Pt mass loadings are summarized in **Table** **1**.

**Table 1:** Denotation and description of Pt/Mo_2_C samples with respective Pt loadings (measured by ICP‐OES)

Denotation	Description	Pt loading [wt%]
Pt_iso_/Mo_2_C	Isolated Pt single atoms	0.67
Pt_quasi_/Mo_2_C	Quasi‐paired Pt single atoms	2.36
Pt_NP_/Mo_2_C	Pt nanoparticles	11.18

The Mo/Zn BIF precursor exhibits a flower‐like morphology assembled from nanoflakes, while the thus‐derived Mo_2_C retains the microflower assembly with additional mesopores in the “petals” (Figure S1, Supporting Information). More structural information of each “petal” is obtained by transmission electron microscopy (TEM). As shown in Figure 1b,c, the Mo_2_C nanoflakes contain mesopores with diameters of around 20 nm, and the lattice fringes show a plane spacing consistent with those of the (200) and (102) planes in *β*‐Mo_2_C. With the 3D self‐assembly of mesoporous 2D nanomeshes, the structure can effectively prevent re‐stacking of Mo_2_C and provide a relatively large surface area (44.7 m^2^ g^−1^, Figure S2, Supporting Information), which is beneficial for well‐dispersing the Pt atoms and facilitating the mass transport of electrolyte ions and oxygen during catalytic reactions. Besides, the Mo atoms can provide strong metal–support interactions with Pt species,^[^ ^23^ ^]^ thus making the Mo_2_C an ideal support for anchoring and stabilizing Pt single atoms, especially for the densely populated (quasi‐paired but non‐contiguous) single atoms explored in the present study. Furthermore, the porous nanomesh morphology of the Mo_2_C support remains stable after impregnating with Pt and subsequent annealing in H_2_, as shown in Figure 1d.

The X‐ray diffraction (XRD) pattern in Figure 1e confirms that the crystal structure of the support material synthesized is consistent with that of the *β*‐Mo_2_C phase, with no impurity phases being observed.^[^ ^24^, ^25^, ^26^ ^]^ The samples with isolated Pt single atoms and quasi‐paired Pt single atoms show no sign of the characteristic Pt peaks, indicating the absence of crystalline Pt phase, which suggests that the Pt could be atomically dispersed on Mo_2_C without agglomerating into larger nanocrystals. For the Pt nanoparticle sample (Pt_NP_/Mo_2_C), small Pt peaks become discernible at the 2*θ* angles of 46.54° and 67.86°, indicating the appearance of crystalline Pt phase (when the Pt loading is increased by nearly fivefold to 11.18 wt%).

## 3 Atomic‐Scale Visualization and Structure Analysis

**Figure 2:**
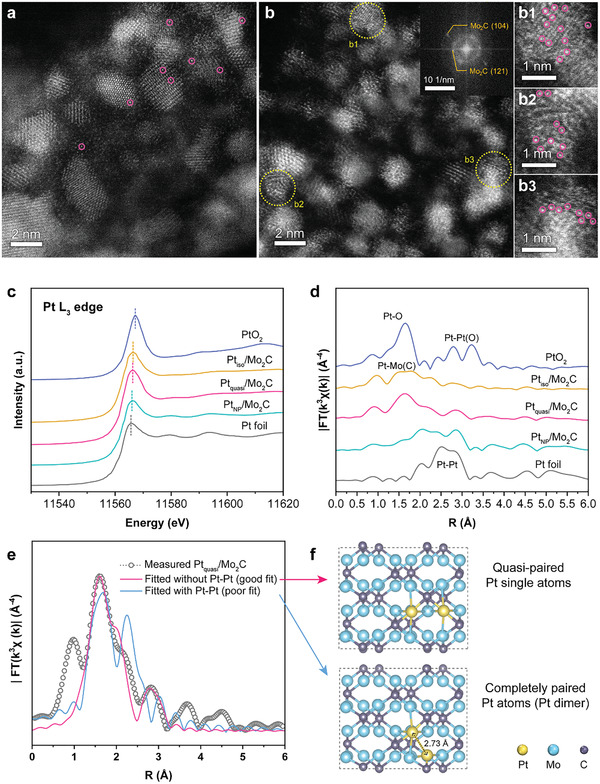
Atomic‐scale insights into the local structure. HAADF‐STEM image of a) Pt_iso_/Mo_2_C and b) Pt_quasi_/Mo_2_C, inset showing the FFT pattern of (b). b1–b3) magnified view of selected regions in (b). c) XANES and d) EXAFS results of samples with different Pt loadings, PtO_2_, and Pt foil. e) Fitted EXAFS results of Pt_quasi_/Mo_2_C, without and with Pt–Pt coordination. f) Structural models of the cases for quasi‐paired Pt single atoms and completely paired Pt atoms (Pt dimers).

The existence of Pt single atoms in Pt_iso_/Mo_2_C and Pt_quasi_/Mo_2_C is further confirmed by high‐angle annular dark‐field scanning transmission electron microscopy (HADDF‐STEM). As shown in **Figure** **2**a,b, Pt single atoms can be distinguished on Mo_2_C substrate as bright dots scattered on the lattice of Mo_2_C (some examples of Pt atoms are indicated by pink circles), which shows no aggregation and proves the ability of the mesoporous Mo_2_C support to stabilize the Pt single atoms. The corresponding fast Fourier transform (FFT) in Figure 2b shows only the characteristics of Mo_2_C, with no indication of any Pt crystal structure, which agrees with the atomic dispersion of Pt. In Pt_iso_/Mo_2_C, the Pt single atoms are scattered farther apart and are isolated from one another, while in Pt_quasi_/Mo_2_C (of which the magnified views of three selected regions are exhibited in Figure 2b 1–b, 3), the atomically dispersed Pt are situated in close proximity with one another, yet without agglomerating into clusters or nanoparticles. As a result of the strong electronic interaction among heterogeneous metal atoms,^[^ ^27^ ^]^ the incorporation of more densely populated Pt single atoms in Pt_quasi_/Mo_2_C causes an apparent distortion in the Mo_2_C lattice. The energy‐dispersive X‐ray spectroscopy mapping of Pt_quasi_/Mo_2_C further corroborates the uniform distribution of Pt element on the Mo_2_C support without signs of aggregation (Figure S3, Supporting Information). In contrast, for Pt_NP_/Mo_2_C with a much higher Pt loading, characteristic signs of Pt nanoparticles can be observed in the STEM images (Figure S4, Supporting Information); the FFT pattern further corroborates the presence of a few Pt crystalline planes, being consistent with the Pt characteristic peaks observed in XRD.

While electron spectroscopy provides the local information, X‐ray absorption spectroscopy (XAS) offers information about the global chemical state and the overall coordination environment of specific atoms, thus serving as a more statistically conclusive indication of the existence of single atoms. As shown in Figure 2c, the Pt L3‐edge X‐ray absorption near edge spectroscopy (XANES) spectra shows the white line positions of the Pt/Mo_2_C samples situate between those of the Pt foil and PtO_2_, suggesting an average valence state of between Pt(0) and Pt(4). Among the three Pt/Mo_2_C samples, the absorption edges of Pt_iso_/Mo_2_C and Pt_quasi_/Mo_2_C shift to more positive energies, suggesting an increase in the oxidation state of Pt for the atomically dispersed Pt. The extended X‐ray absorption fine structure (EXAFS) results further provide information on the coordination environment of Pt. As shown in Figure 2d, the dominant peaks in Pt_iso_/Mo_2_C and Pt_quasi_/Mo_2_C deviate significantly from that of the Pt–Pt coordination. In the heavily loaded Pt_NP_/Mo_2_C, on the contrary, Pt–Pt coordination becomes predominant due to severe aggregation of Pt atoms forming large numbers of Pt–Pt ensembles. In fact, the experimentally measured EXAFS profiles of Pt_iso_/Mo_2_C (Figure S5a, Supporting Information) and Pt_quasi_/Mo_2_C (Figure 2e) fit well with the single atomic configurations, while that of the Pt_NP_/Mo_2_C fits with Pt–Pt coordination (Figure S5b, Table S1, Supporting Information). For the densely loaded Pt_quasi_/Mo_2_C, it is important to confirm that the Pt atoms indeed remain “single”—quasi‐paired and not actually paired (the difference in these two cases being illustrated in Figure 2f), that is, to rule out the existence of immediate Pt–Pt coordination. As such, the fitted EXAFS results of Pt_quasi_/Mo_2_C for the two cases are demonstrated in Figure 2e. A good fit can be obtained for a coordination environment of each Pt being surrounded by four Mo and two C atoms, while introducing Pt–Pt coordination will result in a poor fitting.

X‐ray photoelectron spectroscopy (XPS) is further used to probe the surface chemical states of the elements, and the results can be found in Figure S6, Supporting Information. The Pt 4f_7/2_ peaks for Pt_iso_/Mo_2_C and Pt_quasi_/Mo_2_C shift toward higher binding energies (72.4 eV) as compared to Pt_NP_/Mo_2_C (72.0 eV) and metallic Pt (typically at 71.2 eV^[^ ^28^ ^]^), which further attest to the partially oxidized Pt state in the atomically dispersed Pt catalysts, as a result of the strong metal–support interaction between Pt and Mo_2_C.^[^ ^29^ ^]^

## 4 Electrochemical Performance

**Figure 3:**
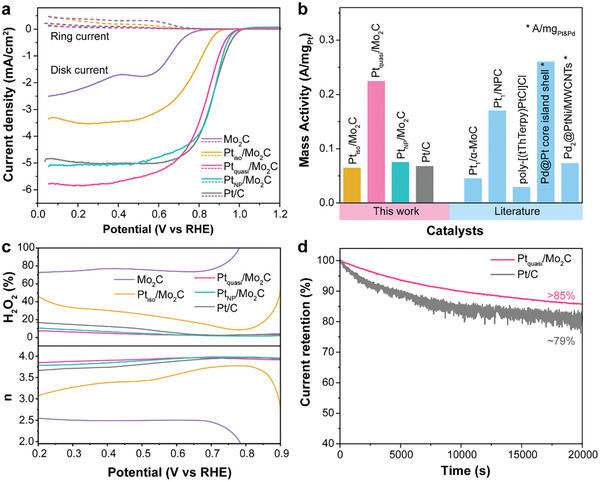
ORR performance. a) LSV, b) mass activity, and c) H_2_O_2_ yield and electron transfer number of the Pt/Mo_2_C samples with different Pt loadings and the commercial (20 wt%) Pt/C (literature mass activity values of typical Pt‐based catalysts are also shown in (b) for comparison. Pt_1_/*α*‐MoC from ref. [30], Pt_1_/NPC from ref. [31], poly‐[(tThTerpy)PtCl]Cl from ref. [32], Pd@Pt core‐island shell from ref. [33], and Pd_2_@PtNi/MWCNTs from ref. [34]). d) Stability of Pt_quasi_/Mo_2_C determined by chronoamperometry test conducted at a potential of 0.85 V.

The ORR performance is evaluated by linear sweep voltammetry (LSV) measurements with a rotating ring‐disc electrode (RRDE) setup at a rotating speed of 1600 rpm, and the results are displayed in **Figure** **3**a. Interestingly, the two samples both with atomically dispersed Pt (Pt_iso_/Mo_2_C and Pt_quasi_/Mo_2_C) demonstrate remarkably different ORR behavior. Pt_iso_/Mo_2_C exhibits much inferior half‐wave potential and diffusion‐limited current density, which is in good agreement with those reported Pt SACs.^[^ ^30^ ^]^ In comparison, the Pt_quasi_/Mo_2_C shows a higher half‐wave potential of 0.83 V, which is approaching the performance of the commercial Pt/C sample (0.86 V) and that of the Pt_NP_/Mo_2_C sample (0.87 V), whilst the latter two have much higher Pt loadings.

The mass activity of each sample is calculated from the kinetic current density at 0.9 V normalized by the respective Pt loading, and the results are depicted in Figure 3b, together with several reported mass activity values that are typical of Pt‐based ORR catalysts. Pt_quasi_/Mo_2_C has a significantly higher mass activity than the other Pt/Mo_2_C catalysts and the Pt/C benchmark. For Pt_NP_/Mo_2_C with a high Pt loading, the atomic dispersion of Pt cannot be maintained, which compromises efficient Pt utilization. Therefore, it is intuitive that the highest mass activity is achieved by a densely populated and yet still atomically dispersed Pt catalyst. On the other hand, when the Pt loading is further reduced, like in the case of the Pt_iso_/Mo_2_C, despite the atomic dispersion of Pt, the 4*e* ORR mass activity also suffers a great loss. This is due to the low areal density of Pt active sites in Pt_iso_/Mo_2_C, that hinders the efficient execution of 4*e* ORR and lowers the intrinsic activity. The effect of low single‐atom density is also evident in the difference in selectivity as analyzed below.

With the RRDE setup, ring current provides information on the yield of H_2_O_2_ during the ORR measurement, and allows the calculation of the electron transfer number (*n*). As shown in Figure 3c, for Pt_iso_/Mo_2_C, there is substantial production of H_2_O_2_ over a wide range of potentials, and the estimated *n* values are between 3.1 and 3.7 over the potential range of 0.2–0.8 V versus reversible hydrogen electrode (RHE). The significant deviation from an *n* value of 4 suggests that selective 2*e* ORR pathway is taking place at a significant proportion for Pt_iso_/Mo_2_C. As it takes two consecutive Pt centers to dissociate the O─O bond and achieve the 4*e* pathway, the 2*e* pathway is normally expected for isolated single‐atom Pt catalysts with minute loadings and Pt sites that are too far apart to act in synergy. At higher Pt loadings—with Pt single atoms getting close enough to become quasi‐paired and acting in synergy, the 2*e* ORR pathway is greatly suppressed, delivering an excellent selectivity toward the 4*e* process via promoting O─O bond cleavage. Indeed, with an electron transfer number of above 3.95 over a wide range of potentials, both Pt_quasi_/Mo_2_C and Pt_NP_/Mo_2_C demonstrate excellent selectivity toward the 4*e* pathway, even outperforming the commercial Pt/C catalyst. To benchmark the catalyst stability with Pt/C, the samples were subjected to chronoamperometry testing, with the potential maintained at 0.85 V and the current response monitored over time. The Pt_quasi_/Mo_2_C sample demonstrated superior stability as compared to Pt/C, with over 85% retention of current density after 20 000 s (Figure 3d).

To demonstrate the efficacy of the single‐atom Pt/Mo_2_C catalyst in practical application, all‐solid‐state zinc–air batteries are assembled with Pt_quasi_/Mo_2_C as the air cathode. For comparison purpose, the performance of the battery with Pt_NP_/Mo_2_C air cathode was also tested. The open circuit voltages of both samples are about 1.36 V (Figure S7a, Supporting Information), which is relatively insensitive to changes in Pt loading. The battery with quasi‐paired single atomic Pt as the cathode demonstrates better reversibility during charging and discharging (Figure S7a,c, Supporting Information), and higher maximum power density (14.86 mW cm^−2^ vs 10.75 mW cm^−2^) as compared to Pt_NP_/Mo_2_C (Figure S7b, Supporting Information), which again testifies to the superior performance of atomically dispersed Pt over its nanoparticulate counterpart. When normalized by the Pt loading, the maximum power density of Pt_quasi_/Mo_2_C can reach 238.14 mW mg_Pt_^−1^, almost six times higher than that of the Pt_NP_/Mo_2_C (40.06 mW mg_Pt_^−1^). To examine the cycling stability, the zinc–air battery devices are subjected to alternating charging and discharging cycles of 20 min each with a constant current density of 1 mA cm^−2^, and the battery device based on Pt_quasi_/Mo_2_C maintains a stable voltage window for more than 1000 min (over 50 cycles), lasting longer than the devices based on Pt_NP_/Mo_2_C (860 min, 43 cycles) and Pt/C (680 min, 34 cycles) (Figure S7c, Supporting Information).

To sum up, the quasi‐paired Pt single atomic catalyst demonstrates a high ORR mass activity, excellent 4*e* ORR selectivity, and superior stability, and demonstrates promise as a zinc–air battery cathode material. The performance enhancements can be attributed to its densely populated and yet stably coordinated Pt single atoms and synergetic actions between the closely neighboring quasi‐paired Pt sites.

## 5 Theoretical Study of ORR Behavior by DFT

**Figure 4:**
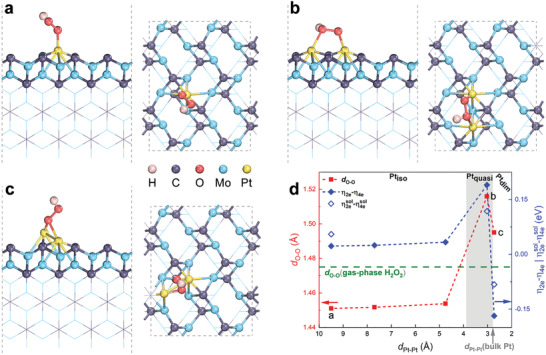
Theoretical analysis of the ORR activity and selectivity. The side and top view of hydroperoxyl adsorption on the Pt site anchored on the 2 × 2 *β*‐Mo_2_C (001) surface with the Pt–Pt distance (*d*_Pt–Pt_) being a) 9.495, b) 3.026, and c) 2.725 Å. The surface structure is displayed by ball and stick models and the rest as line structures. d) The O─O bond length (*d*_O─O_) in the adsorbed *OOH and the difference in overpotential (*η*_2*e*_ − *η*_4*e*_) between the 2*e* and 4*e* ORR pathway as functions of *d*_Pt–Pt_. The overpotential difference obtained with the solvation effect included $\left(\eta_{2e}^{\mathrm{sol}}-\eta_{4e}^{\mathrm{sol}}\right)$ is also shown for *d*_Pt−Pt_ = 9.495, 3.026, and 2.725 Å.

To further elucidate the effect of the mean distance between Pt single atoms on its ORR performance, we carried out first‐principles calculations. Since *β*‐Mo_2_C (001) has been reported as the most stable surface,^[^ ^20^ ^]^ the 2 × 2 supercell of the *β*‐Mo_2_C (001) surface herein has been considered as a representative substrate supporting Pt single atoms with various separations (**Figure** **4**; Figures S8–S12, Supporting Information). As shown in Figure 4a, the supported Pt single atom is coordinated with four Mo and two C atoms, in agreement with our EXAFS results. Note that *OOH (* denotes an adsorbed state) is the common reaction intermediate adsorbate of the 2*e* and 4*e* ORR pathways. An efficient catalyst toward the 2*e* pathway should be capable of preserving the O─O bond in *OOH, whereas for the 4*e* pathway the O─O bond needs to be cleaved efficiently. Thus, we first scrutinized the adsorption of *OOH and its O─O bond length (*d*_O─O_) under different Pt–Pt separations. Figure 4a shows the adsorption of *OOH on an isolated Pt atom, where the minimum distance between two Pt atoms (*d*_Pt–Pt_) is 9.495 Å. Wherein, the *OOH adsorbate is stabilized by forming a vertically oriented single bond on the Pt site. The O─O bond length is found to be 1.451 Å, which is slightly smaller than that in the gas‐phase H_2_O_2_ (1.475 Å). The configuration and O─O bond length keep almost the same when *d*_Pt–Pt_ is shortened from 9.495 to 7.697 and 4.747 Å, as shown in Figure 4d and Figures S9,S10, Supporting Information. However, further decreasing the Pt–Pt distance to 3.026 Å leads to a different adsorption configuration and significant elongation of *d*_O─O_(*OOH) (see Figure 4b,d). In this case, the two Pt single atoms are not bound together but close enough to simultaneously bind one oxygen atom each into Pt─O─O─Pt. As a result, the O─O bond is elongated to 1.516 from 1.451 Å, which is much longer than that in the gas‐phase H_2_O_2_. Such significant O─O bond elongation may suggest that the dispersed Pt atoms at *d*_Pt–Pt_ = 3.026 Å are more selective to the 4*e* pathway than those at larger *d*_Pt–Pt_. For comparison and evaluation of the Pt nanocluster performance, we moved two Pt atoms closer to form a Pt dimer with Pt–Pt chemical bond of 2.725 Å. As illustrated in Figure 4c, the Pt dimer is tilted, and the top Pt atom has a lower coordination compared with the Pt_quasi_ configuration. Here, the *OOH favors a vertical configuration where one oxygen atom binds to both Pt atoms in the dimer. The formation of the Pt─O─Pt ensemble significantly weakens the O─O bond as well (Figure 4d).

After the structural analysis, we investigated the catalytic activity of the supported Pt atom along either the 2*e* or 4*e* pathway through the calculations of the Gibbs free energy under the computational hydrogen electrode model (Figure 4d; Figure S13, Supporting Information).^[^ ^35^, ^36^ ^]^ Figure 4d shows the variation of the difference in the theoretical overpotential (*η*_2*e*_ − *η*_4*e*_) between the 2*e* and 4*e* ORR pathways with respect to *d*_Pt–Pt_. At large Pt–Pt distance (≥4.747 Å), *η*_2*e*_ and *η*_4*e*_ are nearly insensitive to the Pt–Pt distance (Figure S13, Supporting Information) and are comparable (Figure 4d). This is owing to the similar adsorption behaviors (Figures S8–S10, Supporting Information). As the Pt–Pt distance is decreased to 3.026 Å, however, the significant O─O activation causes a higher barrier for reducing *OOH to H_2_O_2_, thereby a higher *η*_2*e*_ but lower *η*_4*e*_ (Figure S13d, Supporting Information). Therefore, the Pt single atom is likely more active toward the 4*e* pathway than the 2*e* pathway at *d*_Pt–Pt_ = 3.026 Å. When the Pt–Pt distance is further decreased to 2.725 Å, the Pt dimer forms, which have a poor catalytic activity toward either the 2*e* or 4*e* pathway (see Figure S13e, Supporting Information). This is because the Pt dimers interact with ORR intermediate adsorbates so strongly that formation and desorption of H_2_O_2_ (H_2_O) for the 2*e* (4*e*) pathway becomes very sluggish. It is worth noting that although including the solvation effect changes the energetics of the reaction intermediates of both 2*e* and 4*e* ORR pathways, the potential‐determining step and the trend in the overpotential difference between the two pathways ($\eta_{2e}^{\mathrm{sol}}-\eta_{4e}^{\mathrm{sol}}$) remains unchanged, as shown in Figure 4d and Figure S13, Supporting Information. Based on these analyses, the experimentally prepared Pt_quasi_/Mo_2_C is very likely associated with the Pt_quasi_ configuration, where the Pt single atoms are not bound to one another but are close enough to interact with the ORR intermediates in synergy, boosting the catalytic performance toward the 4*e* pathway.

By nearing Pt single atoms to a quasi‐paired state, the cleavage of the O─O bond is activated for promoting the four‐electron ORR pathway with high mass activity, exemplifying the promising rewards of synergistic interaction in single atom catalyst systems. In between the cases of isolated single atoms with zero interaction and closely coupled, bond‐forming Pt–Pt dimers, there exists a critical state which we term the quasi‐paired single atoms, that demonstrates fascinating synergy between the closely neighboring and yet non‐contiguous single atomic sites and delivers unique properties in complex catalytic reactions like ORR. The present work serves as a simplified model to study the effect of distance between active single atom sites, and aims to contribute new insights into the design strategies of SACs, by highlighting the significance of the synergistic interactions between neighboring active sites and the evolution of catalytic properties with the degree of separation between the active centers. The attractive ORR properties observed in quasi‐paired Pt single atoms shine light on the possibility to design SACs which exploit the synergistic actions of the neighboring sites and account for spillover of reaction intermediates and hence overcome their intrinsic limitation in multi‐step and multi‐electron catalytic processes.

## 6 Experimental Section

### Chemicals

Ammonium molybdate tetrahydrate ((NH_4_)_6_Mo_7_O_24_·4H_2_O, 99.0%), 2‐methylimidazole (2‐MIM; 99.0%), zinc nitrate hexahydrate (Zn(NO_3_)_2_·6H_2_O; ≥99.0%), and chloroplatinic acid hexahydrate (H_2_PtCl_6_·6H_2_O) were all purchased from Sigma Aldrich and used without further purification.

### Synthesis of Mo/Zn Bimetallic Imidazole Framework

Mo/Zn BIF was synthesized by our reported method.^[^ ^22^ ^]^ In a typical synthesis, (NH_4_)_6_Mo_7_O_24_·4H_2_O (1.96 g, 1.59 mmol) and 2‐MIM (1.30 g, 15.8 mmol) were dissolved into deionized (DI) water (40 mL) in a 100 mL beaker under stirring for 2 h. Next, Zn(NO_3_)_2_·6H_2_O (0.59 g, 1.98 mmol) was added to the above solution followed by further addition of DI water (40 mL). The reaction mixture was stirred for another 4 h and eventually turned from colorless to milky white. Finally, the product was collected by repeated centrifugation and washing with DI water, and dried in a vacuum oven at 70 °C overnight.

### Synthesis of Mesoporous Mo_2_C Support

The Mo_2_C support was obtained by subsequently heating the Mo/Zn BIF at 800 °C and holding for 3 h in 5% H_2_ atmosphere (95% Ar), with a gas flow rate of 300 sccm.

### Synthesis of Pt/Mo_2_C Catalysts

To obtain atomically dispersed Pt on the Mo_2_C support, the IWI method was then adopted.^[^ ^37^, ^38^ ^]^ First, by adding DI water in 5 µL increments to the Mo_2_C powder with vigorous shaking until it just turned into a wet paste, the incipient wetness point of the Mo_2_C powder was determined to be 0.645 mL g^−1^. Then, aqueous solutions of H_2_PtCl_6_·6H_2_O with different concentrations (0.0795 m, 0.3179 m) were prepared and added dropwise to 100 mg of Mo_2_C powder until just reaching its incipient wetness point. With every drop of solution added, the samples were vigorously shaken with a vortex mixer to enable homogeneous mixing. Next, the samples were immediately freeze‐dried and treated in 5% H_2_ atmosphere (95% Ar) at 550 ℃ for 2 h, to obtain atomically dispersed Pt supported on Mo_2_C. The nanoparticle Pt with a higher Pt loading was prepared via a similar route, by impregnating 100 mg Mo_2_C support with 193.5 µL of 0.5 m aqueous H_2_PtCl_6_·6H_2_O solution.

### Characterization

First, the morphology of the materials was characterized by scanning electron microscopy (SEM, SUPRA 40 Zeiss) and TEM (JEOL 2100F). In addition, the N_2_ gas adsorption–desorption measurement was performed to reveal the specific surface area of the material with a Micromeritics 3Flex instrument. Next, the Pt loadings were determined by ICP‐OES (ICP PerkinElmer Optima 5300DV). The crystal structures of the samples were confirmed with XRD by Bruker D8 diffractor using Cu K radiation at 40 kV. Surface bonding nature was analyzed by XPS (AXIS Ultra) measurements. Furthermore, the existence of Pt as single atoms was confirmed by both HADDF‐STEM (JEOL ARM200F) and XAS.

The X‐ray absorption fine structure spectra data were recorded at the 1W1B station of the Beijing Synchrotron Radiation Facility (operated at 2.5 GeV with a maximum current of 200 mA). All samples were pelletized into disks with a diameter of 13 mm and a thickness of 1 mm using BN powder as the binder. All measurements were taken at room temperature. The data acquired were processed and analyzed using Athena software. The first‐shell fitting was processed by Artemis software. For each fitting path, the structural parameters were set to be guessed in the *R*‐range of 1–3 Å.

### Electrochemical ORR Measurements

To prepare the working electrode, 3 mg of Pt/Mo_2_C catalyst and 0.75 mg of carbon black were first dispersed in 1 mL solution containing H_2_O: IPA: Nafion (5 wt%) (in a volume ratio of 2.5:1:0.094) and ultrasonicated for 1 h. 15 µL of the catalyst ink was then drop‐cast onto a glassy carbon RRDE, with a surface area of 0.2475 cm^2^. For comparison, 3 mg of a commercial 20 wt% Pt/C catalyst was dispersed in 1 mL solution containing H_2_O: IPA: Nafion (in a volume ratio of 2.21:1:0.123) and ultrasonicated for 1 h. 10 µL of the Pt/C ink was drop‐cast on the RRDE, corresponding to a Pt loading of 24.24 μg_Pt_ cm^−2^.

The ORR measurements were conducted with a three‐electrode setup using WaveDriver 200 workstation (Pine Research Instruments), in oxygen‐saturated 0.1 m KOH electrolyte, which was continuously bubbled with oxygen during the experiment. The CV measurement and determination of the electrochemical surface area follows the method reported by Garsany et al.^[^ ^39^ ^]^ For each sample, anodic LSV was performed with a scan rate of 5 mV s^−1^ at various rotating speeds. The ORR polarization curves at 1600 rpm were used for comparison across different samples. All potentials were calibrated versus an RHE, according to the equation below.

(1) $$E_{\mathrm{RHE}}=E_{\mathrm{Ag}/\mathrm{AgCl}}\;+0.059\times \mathrm{pH}+0.199\;V$$

The yields of H_2_O_2_ and electron transfer number, *n*, were estimated from the ring and disk currents, according to the following equations:

(2) $$H_{2}O_{2}\left(\%\right)=\;200\times \frac{i_{R}/N}{i_{D}+i_{R}/N}$$

(3) $$n\;=\frac{4\left|i_{D}\right|}{i_{D}+i_{R}/N}\;$$

where *i*_D_ and *i*_R_ are disk and ring current densities, respectively and *N* is the current collection efficiency of the Pt ring disk, which is 0.4 for the instrument used.

### Rechargeable Solid‐State Zinc–Air Battery Tests

In a typical fabrication process, air cathode, Ti mesh current collector, solid‐state electrolyte, and Zn foil were bound in sequence between two pieces of flexible acrylic covers, with an air inlet (1.5 × 0.4 cm^2^) made on the air cathode side by cutting out some portion of the cover. The cathode was prepared by dispersing 7.5 mg of the catalyst, 1.875 mg of carbon black, and 20.3 µL of Nafion in 3 mL ethanol solution followed by ultrasonic treatment to form a uniform dispersion. After that, the dispersion was drop‐cast onto a carbon cloth (size: 1.5 × 0.5 cm^2^) to obtain a mass loading of 3 ± 0.1 mg cm^−2^ and dried at room temperature. To prepare the solid electrolyte, 10 mL of electrolyte consisting of KOH (11.25 mol) and ZnO (0.25 mol) was mixed with 1 g of acrylic acid and 0.15 g of *N, N*′‐methylene‐bisacrylamide, and stirred for 10 min. After removing the white precipitate by filtration, 100 µL of 0.3 m K_2_S_2_O_8_ was added into the electrolyte solution. Once the solution started to polymerize, 350 µL of the electrolyte solution was poured into a 1.5 × 0.5 cm^2^ well cut from a piece of acrylic tape (3M acrylic tape, 1 mm thick). Cycling test was performed using a constant current density of 1 mA cm^−2^. All electrochemical measurements were conducted at room temperature.

### DFT Calculation Details

All calculations were performed based on spin‐polarized DFT as implemented in the Vienna Ab initio Simulation Package (VASP).^[^ ^40^, ^41^ ^]^ The exchange–correlation interaction and the core–electron interaction were described by the Perdew–Burke–Ernzerhof parametrized generalized gradient approximation and the projector augmented‐wave method, respectively.^[^ ^42^, ^43^, ^44^ ^]^ The electronic wavefunction was expanded using a plane wave basis with a kinetic energy cutoff of 500 eV. A 2 × 2 supercell of *β*‐Mo_2_C (001) surface (9.495 Å × 12.118 Å) was adopted to accommodate Pt single atoms (Figure 4). A vacuum layer of around 20 Å was inserted in the out‐of‐plane direction to diminish the spurious interaction between periodically repeated slabs. The convergence criterion of the total energy was set to 1 × 10^−5^_ _eV and the coordinates of the atoms at and above the second bottom carbon layer were fully relaxed until the force on each atom was less than 0.01 eV Å^−1^. For integration over the first Brillouin zone, a Γ‐cantered Monkhorst‐Pack k‐mesh of 5 × 4 × 1 was used. Furthermore, the DFT‐D3 correction method was applied to account for the dispersive van der Waals interaction.^[^ ^45^ ^]^

Under the computational hydrogen electrode model developed by Nørskov et al.,^[^ ^35^, ^36^ ^]^ the Gibbs free energy change for an elementary reaction step of ORR was calculated as follows:

(4) $$\Delta G\;=\;\Delta E+\Delta E_{\mathrm{ZPE}}-T\Delta S+\Delta G_{\mathrm{pH}}+\Delta G_{U}+\Delta G_{\mathrm{sol}}$$

where Δ*E* is the reaction energy. Δ*E*_ZPE_ and Δ*S* are the change in the zero‐point energy and the entropy during the reaction, respectively. *T* is temperature, which is set to 298.15 K. *Δ G_pH_ = −k_B_T × pH × ln 10* is the free energy correction of proton at *pH ≠ 0*, where *k*_B_ is the Boltzmann constant. *Δ GU = −neU*, where *U* is the applied electrode potential and *n* is the number of transferred electrons in the reaction. *ΔG_sol_* is the thermodynamic correction due to the solvation effect, which was calculated using the VASPsol package.^[^ ^46^, ^47^ ^]^ With the calculated Gibbs free energy changes, the limiting potential (*U*_L_), the highest potential at which all elementary reaction steps are downhill in free energy, can be obtained: *U*_L_ = −min Δ*G*_i_. As a result, the theoretical overpotential (*η*) can be calculated using *η*  = *U*_eq_ −*U*_L_. *U*_eq_ is the equilibrium potential, which is 1.23 V for *O_2_ + 4(H^+^ + e^−^) → 2H_2_O* and 0.7 V for *O_2_ + 2(H^+^ + e^−^) → H_2_O_2_* at standard conditions (pressure *p*  =  1 bar, pH  =  0 and *U*  =  0 V), respectively. Note that according to the Nernst equation *U*_eq_ has the same dependence on pH as *U*_L_. Thus, *η* is independent of pH and Δ*G* at standard conditions is herein presented.

## Conflict of Interest

The authors declare no conflict of interest.

## Supporting information

Supporting Information

## Data Availability

The data that support the findings of this study are available from the corresponding authors upon reasonable request.
