# Supplementary material for: Quasi‐Paired Pt Atomic Sites on Mo2C Promoting Selective Four‐Electron Oxygen Reduction
Source: Adv Sci (Weinh). 2021 Jul 14;8(18):2101344. doi: 10.1002/advs.202101344 (PMC8456204; doi:10.1002/advs.202101344)
Supplement: Supplementary file 1 — Supporting Information [file ADVS-8-2101344-s001.pdf]

WILEY-VCH

© 2021 Wiley-VCH GmbH

ADVANCED SCIENCE
Open Access

Supporting Information

for *Adv. Sci.,* DOI: 10.1002/advs.202101344

Quasi-paired Pt atomic sites on Mo2C promoting selective four-electron oxygen reduction

Lei Zhang, Tong Yang, Wenjie Zang, Zongkui Kou, Yuanyuan Ma, Moaz Waqar, Ximeng Liu, Lirong Zheng, Stephen J. Pennycook, Zhaolin Liu, Xian Jun Loh, Lei Shen*, John Wang**

WILEY-VCH

© 2021 Wiley-VCH GmbH

Supporting Information

Quasi-paired Pt atomic sites on Mo_2C promoting selective four-electron oxygen reduction

Lei Zhang, Tong Yang, Wenjie Zang, Zongkui Kou, Yuanyuan Ma, Moaz Waqar, Ximeng Liu, Lirong Zheng, Stephen J. Pennycook, Zhaolin Liu, Xian Jun Loh, Lei Shen*, John Wang**

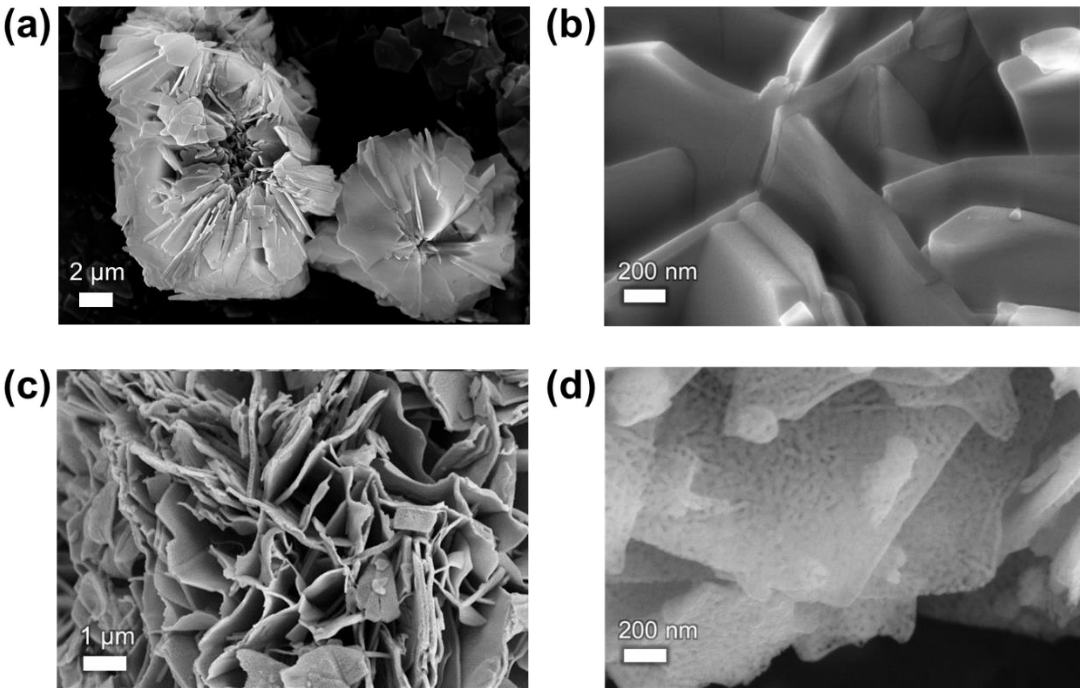

Figure S1. Morphology of the precursor and Mo_2C catalyst support. SEM images of a-b) Mo/Zn BIF and c-d) β-Mo_2C support.

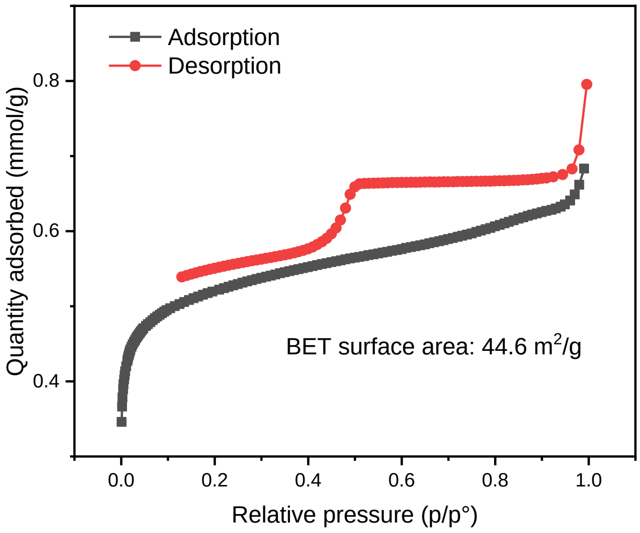

Figure S2. N_2 adsorption-desorption isotherms of the β-Mo_2C support.

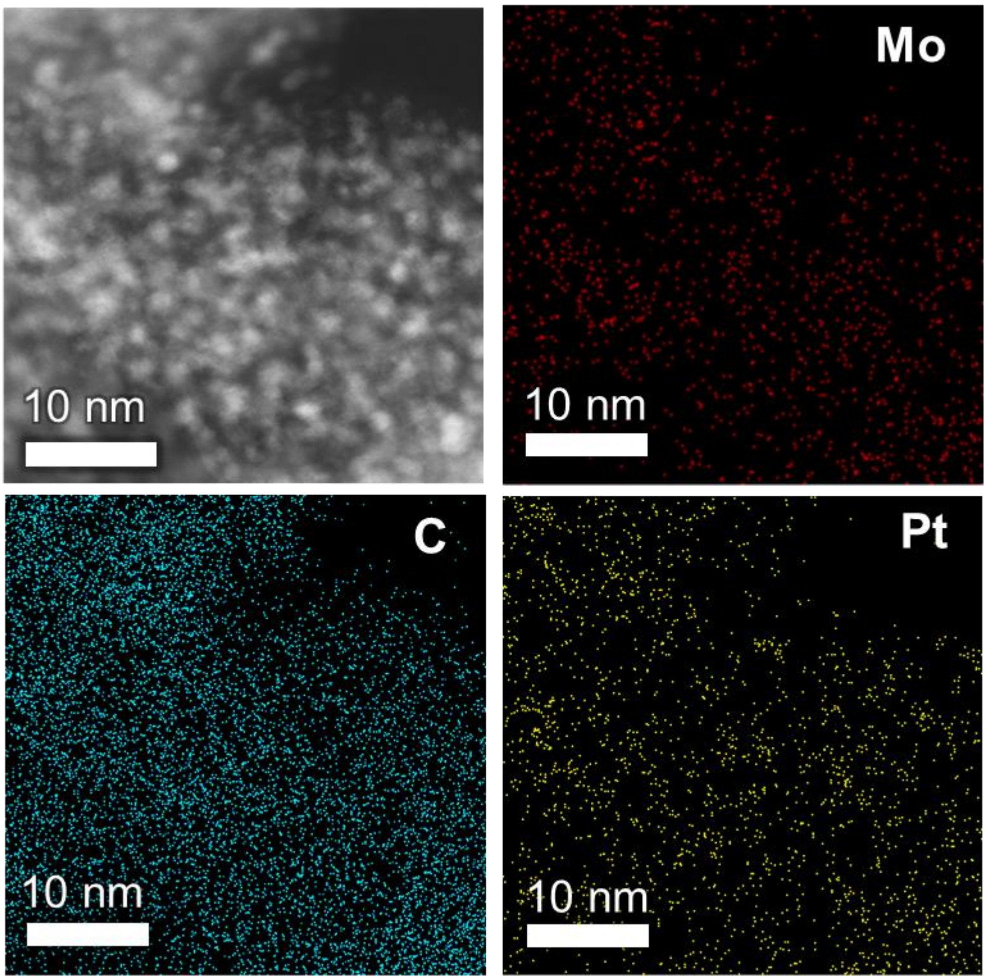

Figure S3. The EDS mapping result of Mo, C, and Pt elements are shown for a representative region in the Pt_{quasi}/Mo_2C catalyst, showing uniform distribution of Pt on the Mo_2C support.

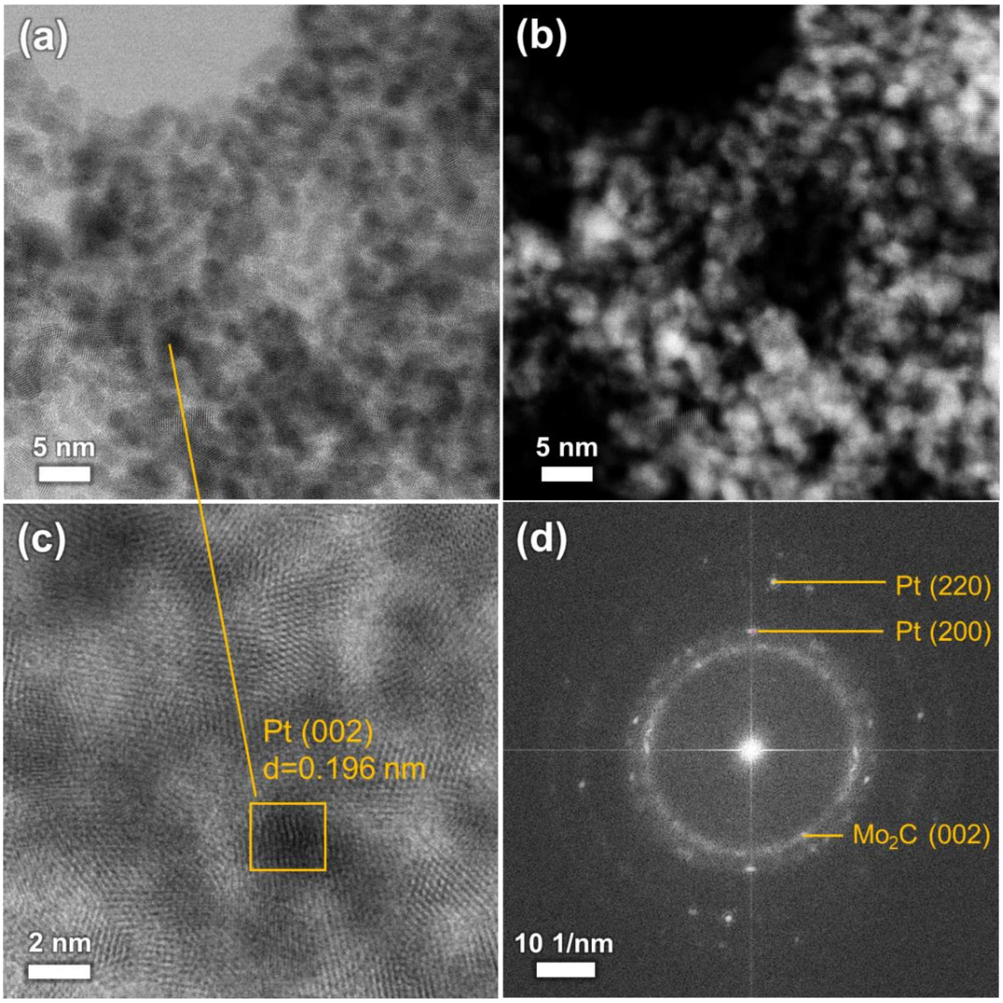

Figure S4. Morphology and structure characterization of Pt_{NP}/Mo_2C. a-c) STEM images of Pt_{NP}/Mo_2C. a) Bright-field and b) high-angle annular dark-field (HADDF) images of the Pt_{NP}/Mo_2C, with c) showing a magnified region in (a). d) Fast Fourier Transform (FFT) of (a), showing crystal spacings from both Mo_2C and Pt.

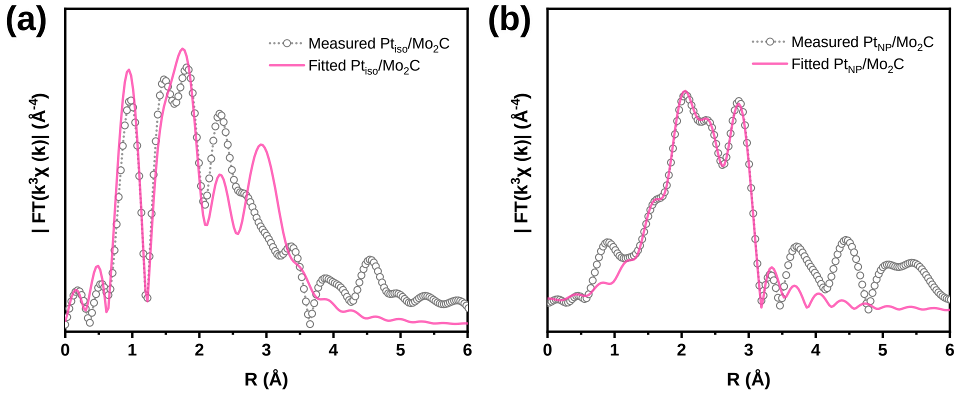

Figure S5. Fitted EXAFS results of a) Pt_{iso}/Mo_2C and b) Pt_{NP}/Mo_2C, with detailed fitting parameters compiled in Table S1. The models for the EXAFS fitting of Pt_{iso}/Mo_2C and Pt_{NP}/Mo_2C correspond to the structures presented in Figures S8a and S12a, respectively.

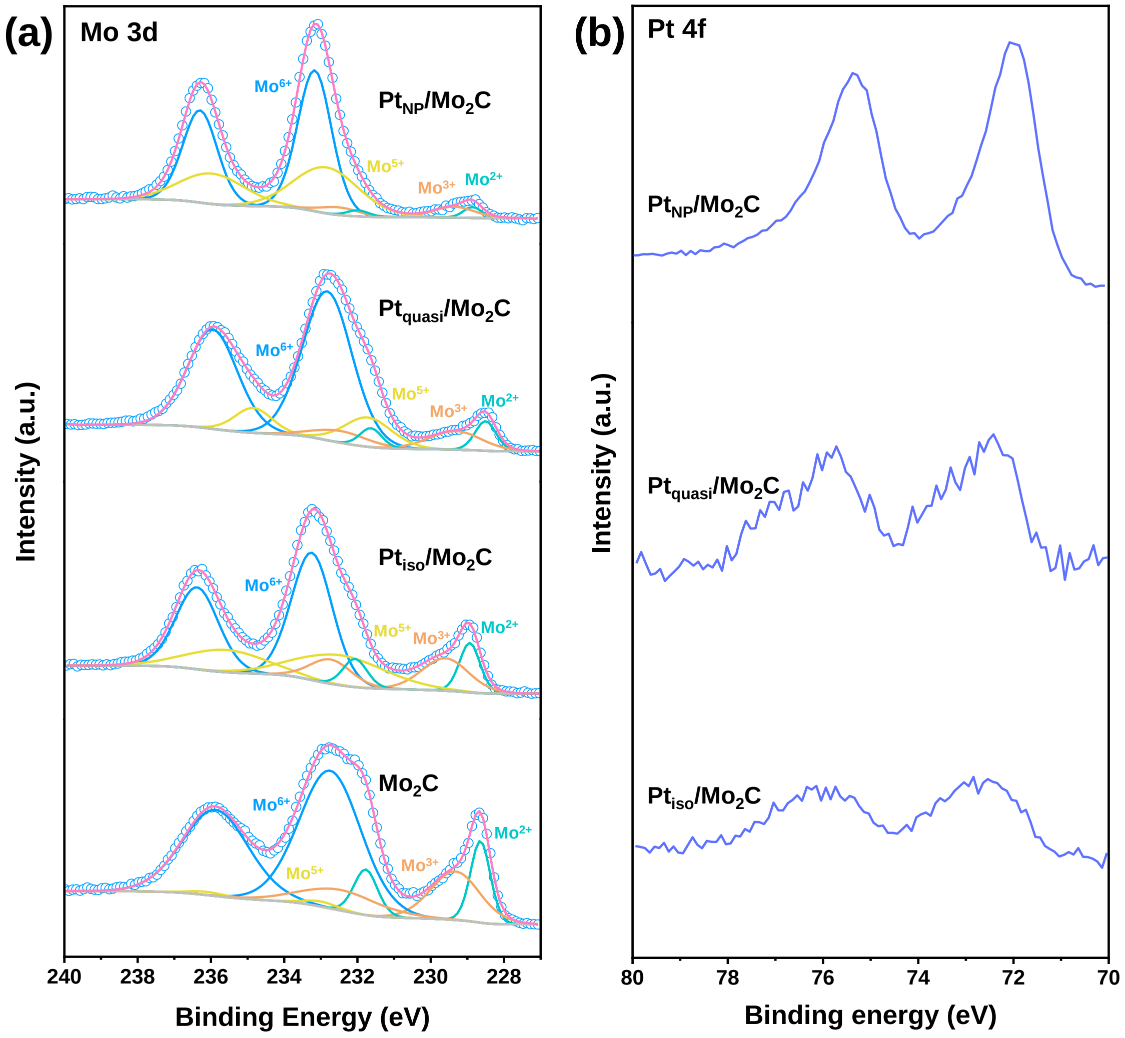

Figure S6. Chemical state characterization by XPS. a) Mo 3d and b) Pt 4f XPS results of various Pt/Mo_2C samples.

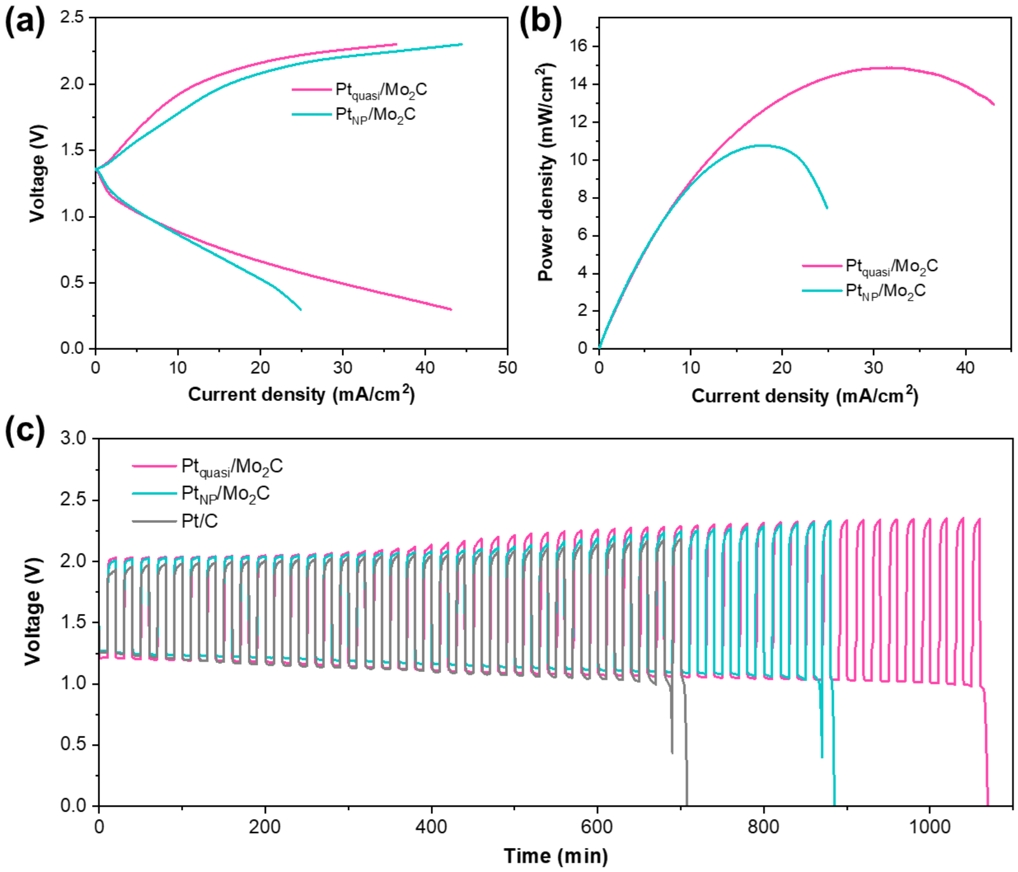

Figure S7. Performance of solid-state Zn-air batteries assembled from Pt$_{quasi}$/Mo$_2$C and Pt$_{NP}$/Mo$_2$C. a) Charge and discharge polarization curves; b) power-current density curves; c) cycling stability measured with alternating charging and discharging cycles of 20 minutes each and a constant current density of 1 mA cm^{-2}.

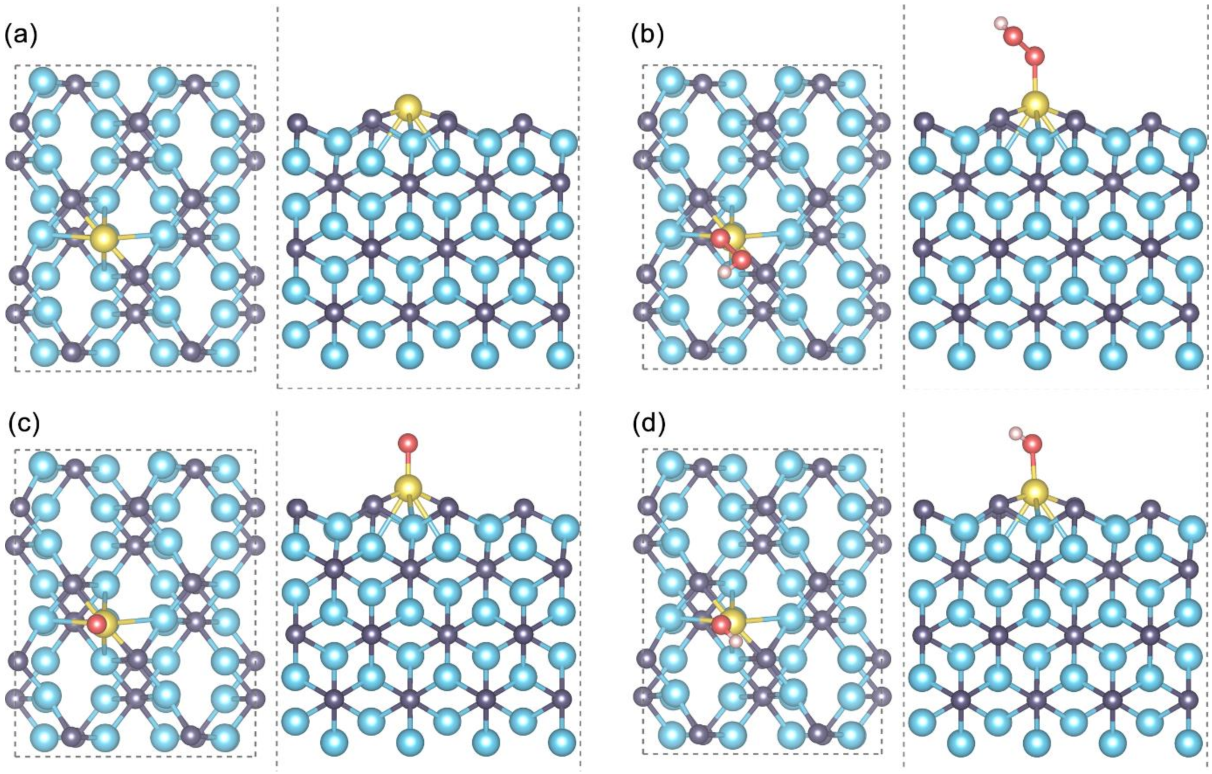

Figure S8. The top and side view of *β*-Mo_2C (001) supported Pt single atoms with the minimum Pt-Pt distance being 9.495 Å. a) the clean surface. b-d) the adsorption of b) *OOH, c) *O and d) *OH, respectively. * denotes an adsorbed state.

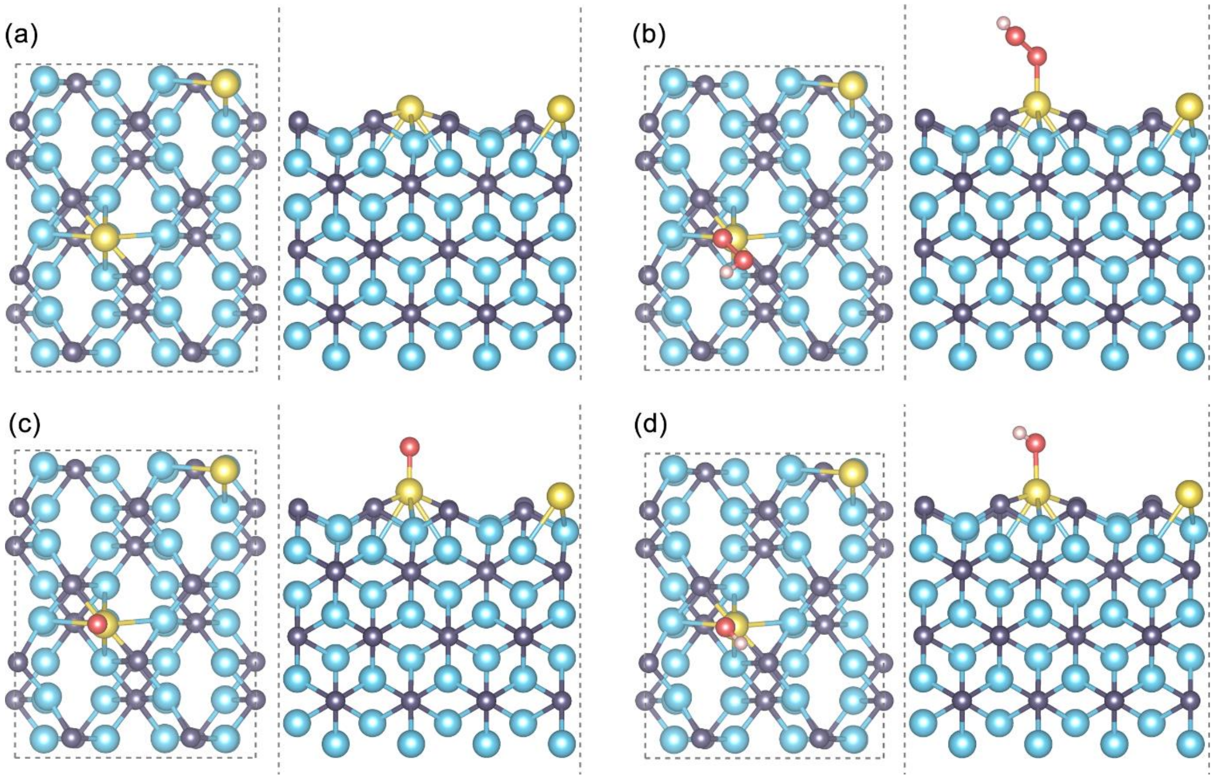

Figure S9. The top and side view of β-Mo_2C (001) supported Pt single atoms with the minimum Pt-Pt distance being 7.697 Å. a) the clean surface. b-d) the adsorption of b) *OOH, c) *O and d) *OH, respectively. * denotes an adsorbed state.

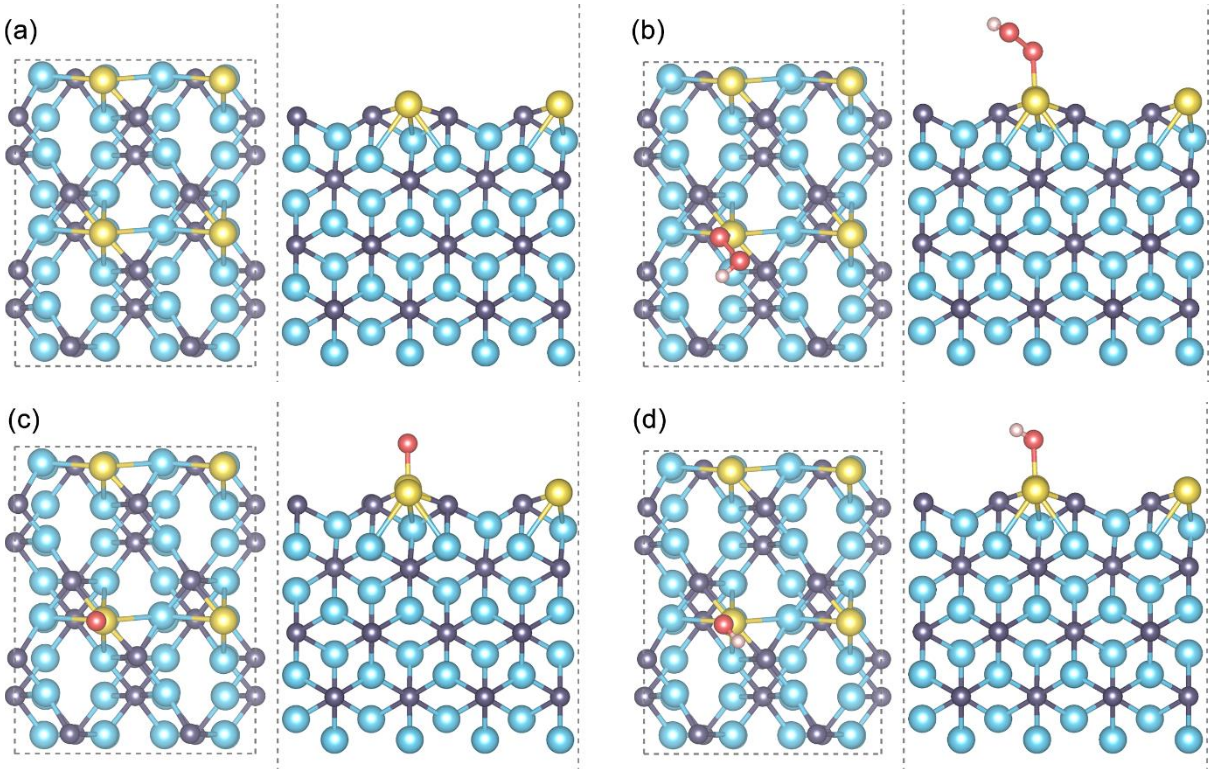

Figure S10. The top and side view of β-Mo_2C (001) supported Pt single atoms with the minimum Pt-Pt distance being 4.747 Å. a) the clean surface. b-d) the adsorption of b) *OOH, c) *O and d) *OH, respectively. * denotes an adsorbed state.

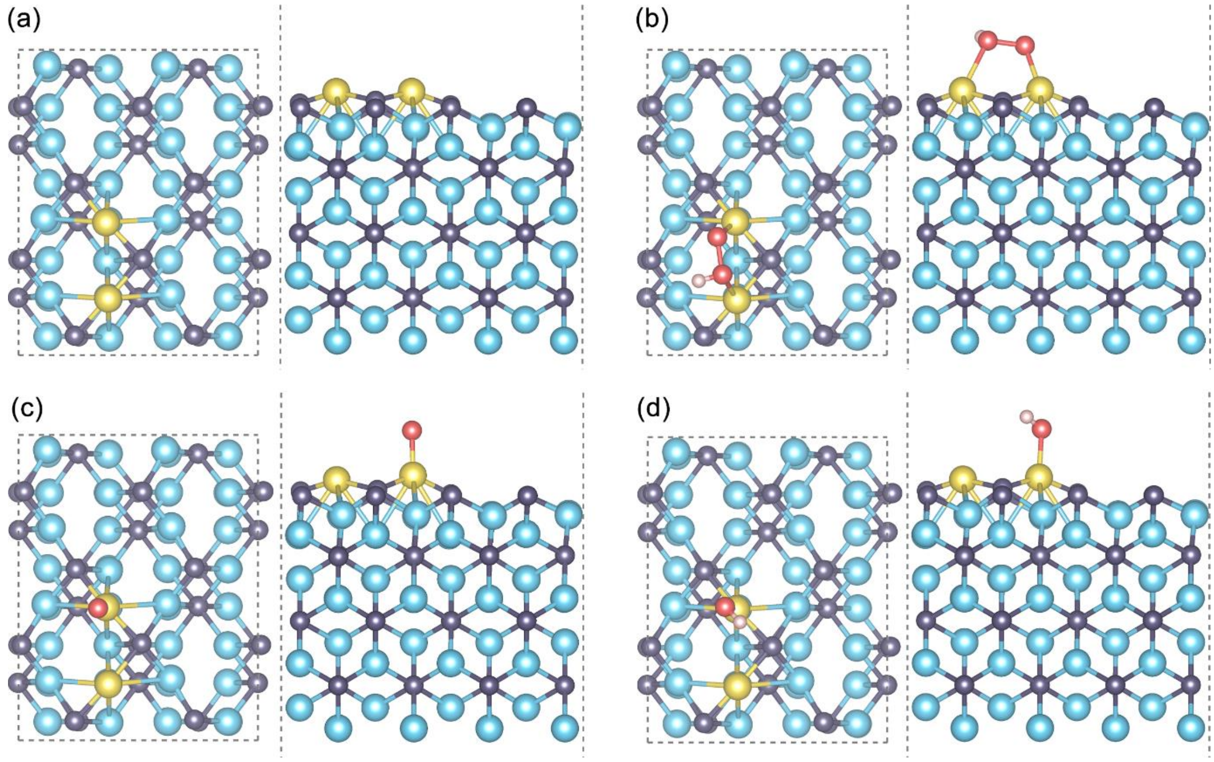

Figure S11. The top and side view of β-Mo_2C (001) supported quasi-paired Pt single atoms with the Pt-Pt distance being 3.026 Å. a) the clean surface. b-d) the adsorption of b) *OOH, c) *O and d) *OH, respectively. * denotes an adsorbed state.

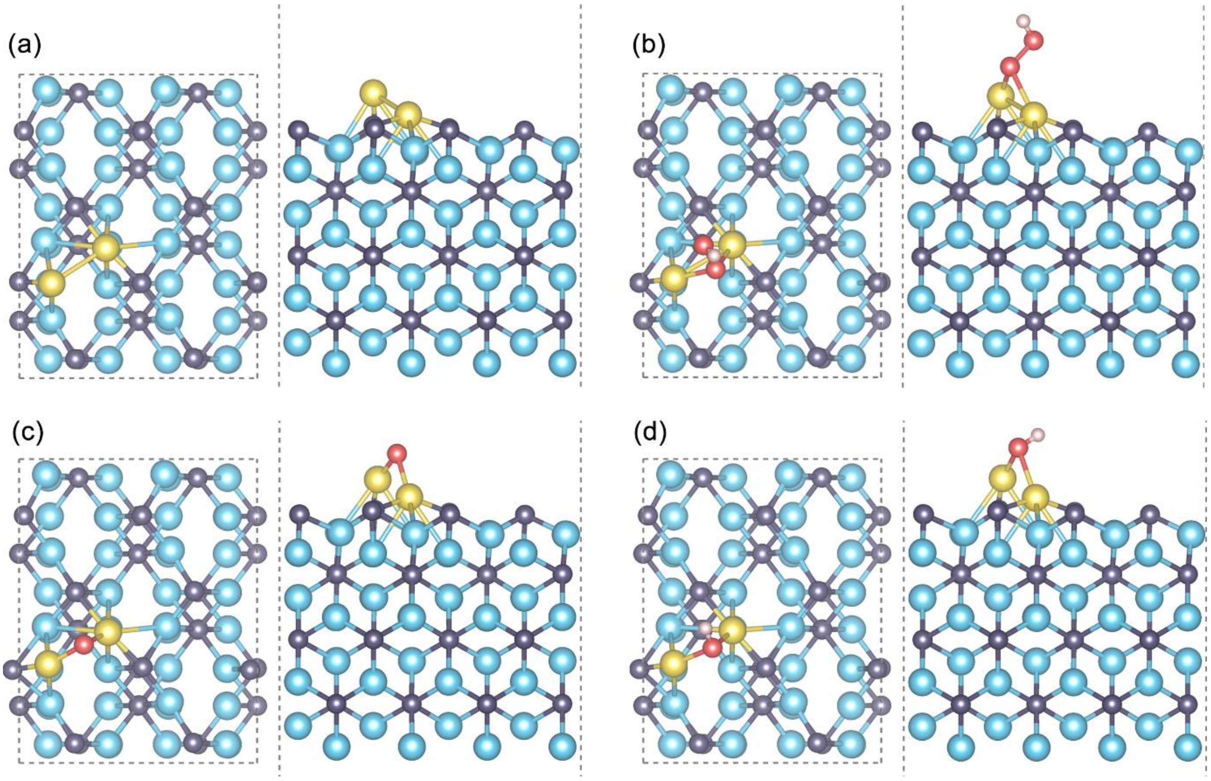

Figure S12. The top and side view of β-Mo_2C (001) supported Pt dimers with the Pt-Pt distance being 2.725 Å. a) the clean surface. b-d) the adsorption of b) *OOH, c) *O and d) *OH, respectively. * denotes an adsorbed state.

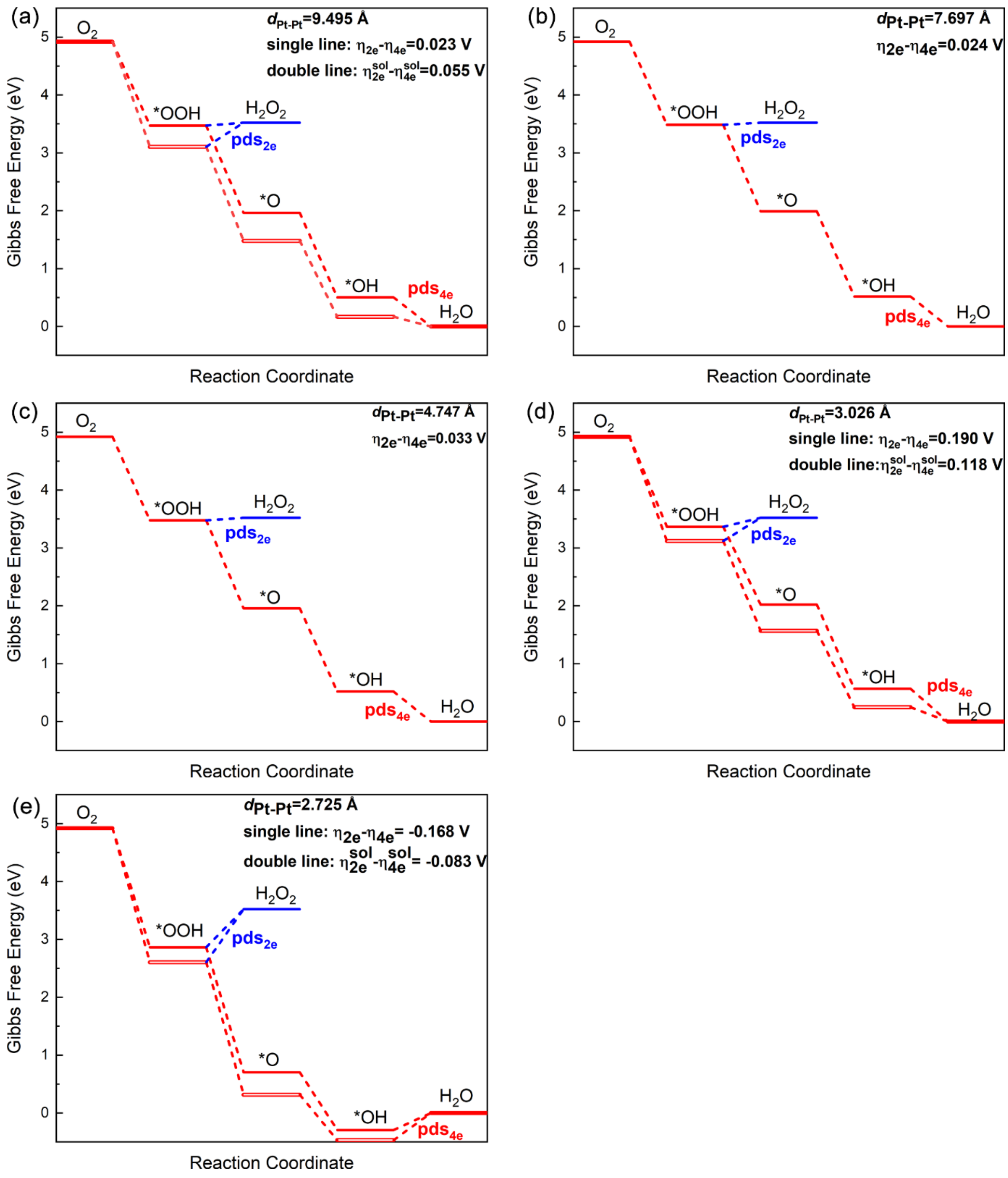

Figure S13. The free energy diagram of oxygen evolution reaction along the 4*e* and 2*e* pathways. The free energies were calculated on the stabilized Pt site with the minimum Pt-Pt distance (d_{Pt-Pt}) being a) 9.495 Å, b) 7.697 Å, c) 4.747 Å, d) 3.026 Å and e) 2.725 Å. The potential-determining step (pds) of the 4*e* and 2*e* pathways and the overpotential difference (η_{2e}- η_{4e}) are marked. Panels a), d) and e) also illustrate the free energy diagram obtained with the solvation effect included (double line). The corresponding overpotential is denoted as

$\eta_{2e}^{sol} - \eta_{4e}^{sol}$.

Table S1. Pt L3-edge EXAFS fitting results for Pt/Mo_2C catalysts

Catalyst	Shell	Bond length [Å]	C.N.	σ^2 [$Å^2$]	R factor
Pt_{NP}/Mo_2C	Pt-Pt	2.71	7.4	0.00818	0.0026
	Pt-Mo	3.21	0.9	-0.00592	
	Pt-O	1.97	0.6	0.01033	
Pt_{iso}/Mo_2C	Pt-Mo	2.96	4.99	0.01773	0.0416
	Pt-C	1.48	0.88	0.00111	
	Pt-O	2.08	2.05	0.00918	
Pt_{quasi}/Mo_2C	Pt-Mo	2.86	3.91	0.01618	0.0167
(Good fit)	Pt-C	2.02	1.76	0.00391	
	Pt-O	2.22	1.55	0.01071	
Pt_{quasi}/Mo_2C	Pt-Mo	2.87	3.91	0.01606	0.0834
(Poor fit)	Pt-C	2.24	1.76	0.00864	
	Pt-O	1.97	1.55	0.00571	
	Pt-Pt	2.62	1.00	0.00300	
